# Visibility and Analytics Network (VAN) approach to improve immunization supply chain and management performance system in Pakistan

**DOI:** 10.7189/jogh.11.06002

**Published:** 2021-03-07

**Authors:** Arshad Altaf, Anees Siddiqui, Agha Muhammad Ashfaq, ASM Shahabuddin

**Affiliations:** 1 Frontiers in Public Health Research and Education, Karachi, Pakistan; 2 Expanded Programme on Immunization (EPI), Health Department Sindh, Karachi, Pakistan; 3 Implementation Research and Delivery Science Unit, Health Section, Programme Division, United Nations Children's Fund, New York, New York, USA

## Abstract

**Background:**

Pakistan has been experiencing several immunization related challenges. The supply chain management information system (MIS) is considered an important component of immunization services as it can improve visibility in key areas such as vaccine shortages or wastage. This study assessed the effectiveness of the Visibility and Analytics Network (VAN) approach in improving vaccine supply and availability of stocks by comparing the situation in intervention and non-intervention districts in Sindh province of Pakistan.

**Methods:**

We utilized a quantitative and qualitative approach to collect data to assess the VAN approach in two districts of Sindh province in Pakistan. The data were collected between August and October 2017. VAN is a systematic monitoring system which measures the performance of vaccine supply chain management based on a set of indicators. We assessed storage facilities of the Expanded Programme on Immunization (EPI) in Sindh and interviewed personnel involved using a pre-tested data collection tool. We also conducted in depth interviews with senior management to assess performance of VAN, adoption mechanism and needs to scale up the VAN approach.

**Results:**

We assessed 52 EPI facilities of Sindh province government. In the intervention district 83.3% managers were using MIS data for decision making related to vaccine supplies whereas in the non- intervention district no MIS based data were available. Ninety percent of stores were maintaining a stock registry and 100% supplies matched with requisitions in the intervention district compared to 40% and 35% in the non-intervention district for the same variables. Vaccine wastage was high in the non- intervention district (BCG 46.7% vs 33.9; OPV 13.5% vs 9.5%; pneumococcal 11.4% vs 7.4%). In-depth interview findings suggested that the VAN approach provided data guided monitoring in Pakistan for the first time. The approach also enabled district managers to make timely decisions.

**Conclusion:**

The VAN approach improves vaccine supply chain management. It should be scaled up and implemented at national or sub national especially in countries struggling with vaccine supply chain management.

The World Health Organization (WHO) estimates that more than 50% vaccines are wasted each year due to multiple reasons some of which are logistics and shipment related and also because of poor temperature control [1]. A survey in 2016-17 conducted in two districts of India (Pune and Kangra) reported high wastage rates for all vaccines. In Kangra the wastage rates for different types of vaccines were: BCG 37.1%, DPT 32.1%, Measles 32.2%, OPV 50.8%, TT 34.1% and pentavalent 18.4%. While in Pune district the reported rates for vaccines wastage were: BCG 35.1%, DPT 25.4%, Measles 21.7%, OPV 14.3%, TT 23.1% and pentavalent 13.2% [2]. To assess missed opportunities for vaccination, the vaccine wastage rates for different categories were assessed in Cambodia and the assessment had found in 100 randomly selected health facilities in 24 districts the wastage rate for single dose vaccine was 4% while for multiple dose vaccines it was as high as 60% [3]. The vaccine wastage rates were observed in a prospective study in Gambia and it was reported that wastage rates for the lyophilised vaccines BCG, Measles and Yellow Fever ranged from 18.5%-79.0%, 0%-30.9% and 0%-55.0%, respectively, mainly through unused doses at the end of an immunization session [4]. In adequate vaccine supply chain management was cited as one of the reasons for vaccine wastage in these countries. Wastage of vaccine due to overestimation in procurement has also been documented from Pakistan [5]. Pakistan faces many immunization-related challenges and efforts to increase immunization coverage have been ongoing at national and international levels [6-10]. A plan of action developed based on a literature review of public health and polio issues concluded that having a programmatic, system-wide, socio-cultural and ethical approach is needed for policy makers and the programme managers in Pakistan to address the multitude of barriers to polio vaccination [11]. A Cochrane review published in 2016, in which four studies from Pakistan were selected, concluded that improved service provision may improve childhood immunization coverage [12].

In many settings around the world the storage and transportation of vaccines is challenging, with most vaccines having to be kept consistently at a temperature between 2°C and 8°C. Ineffective supply chain management can lead to vaccines being wasted or losing their potency [13-15]. WHO’s advisory committee recommended in 2014 that immunization programmes measure and invest in their supply chain management system and implement improvements. The advisory committee also called upon the global community of partners to raise awareness and investments to harmonize supply chain management systems [16].

The United States Agency for International Development (USAID) in 2013 supported a supply chain and logistical management project to expand the web-based Logistics Management Information System (LMIS) to include a supply chain logistics management system [17]. The project enhanced the system to strengthen the logistics and inventory management of vaccines in a comprehensive manner, including adapting the existing Vaccine Logistics Management Information System (vLMIS) (http://v.lmis.gov.pk) and global standard 1 (GS1) barcoding to meet the needs of vaccines and related commodities (eg, diluents and syringes) as well as cold chain equipment [18]. The Pakistan vLMIS project used a Visibility and Analytics Network (VAN) approach to enhance data visibility, analysis and action for the immunization supply chain by integrating principles and quality improvement processes into existing team structures at the national level and in Sindh province in three pilot districts [17]. The VAN approach has built capacity of people at each level, defined processes for routine data analysis and action (quality improvement), and refined technology to ensure that the correct data are selected and displayed at each level to enable effective analysis and appropriate action [17]. It has been demonstrated elsewhere that when the quality improvement teams (QITs) meet regularly to review and use data for decision making, significant improvements can be observed in supply chain performance [19]. Early lessons learned from country VAN experience in Tanzania, Ethiopia, Kenya and Pakistan showed that the approach was helpful at an early stage in identifying gaps in data dashboards [19].

Our study assessed the effectiveness of the VAN approach in improving the vaccine supply and availability of stocks at service delivery points (SDPs) by comparing the situation in intervention and non-intervention districts in Sindh province of Pakistan. It also explored enabling factors and barriers in the roll out of the VAN approach.

## METHODS

We utilized a quantitative and qualitative approach to assess the VAN approach in two districts of Sindh province in Pakistan. Data were collected between August and October 2017. Ghotki district was the VAN-intervention district and Sukkur was the non-intervention district. These districts were selected because of their similar socio-demographic indicators, close vicinity and almost similar number of union councils (UCs). We evaluated storage facilities of Expanded Programme on Immunization (EPI) and interviewed personnel involved in data entry using a pre-tested data collection tool that contained questions related to immunization supply chain management. We conducted in-depth interviews with key informants who were senior management personnel of the VAN project.

### Study setting

The study was conducted in two districts of Sindh Province, Ghotki and Sukkur. The two districts have several similarities: they are close to each other and the size of the population is almost the same, with Ghotki having an approximate population of 1.6 million and Sukkur’s population is 1.4 million, according to the latest Pakistan Demographic Survey (2017); and cultural and traditional practices resemble each other. Both districts have an agricultural economy with the slight difference that Sukkur is also a trading hub due to its central location in the province. Cotton and rice are two of the main crops of this region. Brick making is a common practice in the plains of both districts. According to January to August 2018 vLMIS data, the EPI coverage rate for Ghotki was 64.1%%and for Sukkur it was 57%.

### VAN approach

The VAN approach relies on a framework that is based on a set of indicators which are used systematically to assess the performance of vaccine supply chain management [20]. The broad indicators defined elsewhere [20] were:

- MIS training status of district manager and support staff,
- MIS data used for vaccine supply chain decision making,
- Stock status of each vaccine verified by reviewing registers and data,
- Quality of record keeping (eg, maintained stock register, vaccine tags, receipt vouchers),
- Matching of supply with requisite quantity, and
- MIS reporting regularity in the past six months.

### Descriptions of supply chain related facilities evaluated

***District stores***: These are medium- to large-size storage facilities in each district. Vaccine supplies are delivered here from the Divisional Warehouse in Sukkur. No immunization services are offered here (this is for storage only).

Service delivery points (SDPs)/EPI centres: These are the EPI centres where immunization services are provided, or vaccinators take the vaccines and supplies from SDPs to go into the field.

Taluka or tehsil (sub-district) stores: Most of these stores are housed in Taluka Headquarter hospitals. The designated EPI staff from the Service Delivery Points (SDPs) collect supplies from the *taluka* or *tehsil* stores.

### Quantitative component of the study

#### Study sample

The study sample consisted of vaccine supply stores in the two districts and their sub stores. In both districts, the main supply stores were included. Besides the main stores in Ghotki, sub-district stores in five *talukas* were included as well. Similarly, in Sukkur sub stores in five *tehsils* were also included. The SDP stores were located throughout each district. In Ghotki, there were 53 SDPs out of which 20 were randomly selected and evaluated. In Sukkur there were 63 SDPs and 20 were randomly selected and assessed. A proper sample-size calculation was not performed because of the small number in total. The number 20 was selected because it was almost half the size of all facilities and these were randomized to keep an element of generalizability. For the randomly selected stores, if the selected store was found to be unavailable at the day of the visit the next nearest one was selected to replace it.

#### Data collection tool

We used a pre-tested supply chain management assessment tool that had quantitative and semi-structured questions. The tool was based on data collection instruments used in the final evaluation report of Delivery Project [17]. The project team reviewed the first two drafts then it was piloted along with a UNICEF appointed focal person in the District Health Office of Sukkur, Pakistan. During the piloting process the flow, placement and relevance of questions was the primary objective. After three rounds of iteration the study tool was finalized.

The quantitative questions focused on the following:

1. Details of implementers and investigators,
2. Details of store from where data were collected,
3. Details of team collecting data,
4. LMIS utilization,
5. Stock status (details of each vaccine and related supplies, stock register, physical count, status of stock, etc.),
6. Details of how the record is maintained,
7. Regularity of MIS reporting,
8. Usage of USAID-supplied hardware,
9. Semi-structured questions used as process indicators.

#### Data management

All quantitative data were collected on printed questionnaires, which were in MS Excel format. Two team members who had participated in the project training and collected all of the quantitative data in the field. After collecting the information and manually verifying stocks, data collectors rechecked the same questionnaire for any missing information and completed it the same evening in order to ensure that nothing was missed or incorrect. All data were entered in MS Excel which was also used for generating tables and graphs.

### Qualitative component of the study

Key informant interviews were planned keeping in mind the change process. No theoretical framework was used to develop the in depth interview tool. The key informants were selected based on a collective decision and their roles in decision making at macro level. Interviews were conducted with provincial leadership of EPI, and the provincial and federal level senior management of the Deliver Project as key informants.

The qualitative questions used for in-depth interviews of key informants had three key sections:

1. Performance assessment of QIT/VAN model,
2. Adoption mechanisms,
3. Support system required for scaling up.

Interviews of key informants was conducted by one of the team members (AA). Data for key informant interviews was analyzed manually and organized thematically considering the objectives of this study. No software was used for this purpose.

### Ethical clearance

The proposal was reviewed, and ethical clearance was obtained from the Institutional Review Board of the Health Services Academy, Pakistan, prior to collecting any data. Verbal consent was obtained from all participants prior to interview.

## RESULTS

A total of 52 of Sindh EPI’s logistics and management facilities were evaluated in this study. Out of these, one in each district was the main supply store, five in each district (10 altogether) were *taluka* stores, and 20 from each district (40 altogether) were SDPs in the EPI centres. In the 12 EPI storage facilities in both districts (two main district and 10 sub-district facilities), there was marked difference between the processes in the intervention and non-intervention districts. For example, in Ghotki, the intervention district, 83.3% managers were using MIS data for decision-making related to vaccines and supplies; whereas no MIS-based data was available in the non-intervention Sukkur district. In the intervention district, the percentage of supply that matched with requisitioned from quantities was 83.3%, while in the non-intervention district this match was 33.3%. Working hardware was 100%in the intervention district compared to 50%in the non-intervention district.

In the SDPs (n = 40), in the intervention district 90% of the stores were maintaining stock registers compared to 40%in the non-intervention district (**Table 1**). Receipt vouchers were maintained in 100% of the facilities in Ghotki and in Sukkur 60% of the SDPs were maintaining receipt vouchers. The proportion of SDPs where the supply matched with the requisitions from quantities was 100% in Ghotki compared to 35% in Sukkur. Stock outs were reported from 10% of the SDPs in the intervention district compared to 40% in the non-intervention district. **Table 1** provides comparison of logistics at various service delivery points. In the intervention district (Ghotki) the situation is far better compared to the non-intervention district (Sukkur).

**Table 1:** Description of logistics at service delivery points (SDPs) (n = 40)

Variables	Ghotki intervention district (n = 20)	Sukkur non-intervention district (n = 20)
Number of SDPs with stock outs	2 (10%)	8 (40%)
Number of SDPs maintaining stock registers	18 (90%)	8 (40%)
Number of SDPs maintaining issue receipt vouchers	20 (100%)	12 (60%)
Number of SDPs whose supply matched with requisitions from quantities	20 (100%)	7 (35%)

### Status of one-month antigen stock

**Figure 1:**
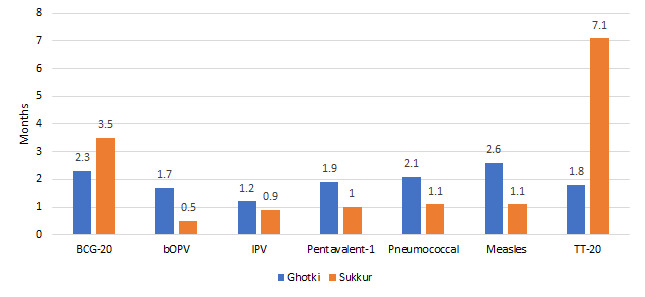
Status of vaccine stock in the intervention (Ghotki) and non-intervention (Sukkur) districts. EPI benchmark for stock: Over stock >1.5 months, Adequate = 1-1.5 months, Under stock = 0.5-1 month, Stock out = 0.

The stock data for the month of August 2017 was collected and reviewed for the intervention and non-intervention districts (**Figure 1**). If any store had stock of more than one and a half months that was considered as ‘over stock’ by EPI and is a negative performance indicator. Stock of less than one month is also taken as a negative performance indicator. The district store in Sukkur had OPV and IPV for less than one month while Pentavalent 1, Pneumococcal and Measles were available for one or 1.1 months. Sukkur was overstocked for BCG and tetanus toxoid. The district store in Ghotki was slightly overstocked for some antigens but it was not understocked.

### Wastage rates

The vaccine wastage rate is a key indicator of supply chain management. EPI allowable wastage rates for each antigen are:

- BCG vaccine – 50%,
- Bivalent oral polio vaccine (bOPV) – 20%,
- Inactivate polio vaccine (IPV) – 50%,
- Pentavalent vaccine – 5%,
- Pneumococcal vaccine – 10%,
- Measles vaccine – 20%,
- Tetanus toxoid – 20%.

**Figure 2:**
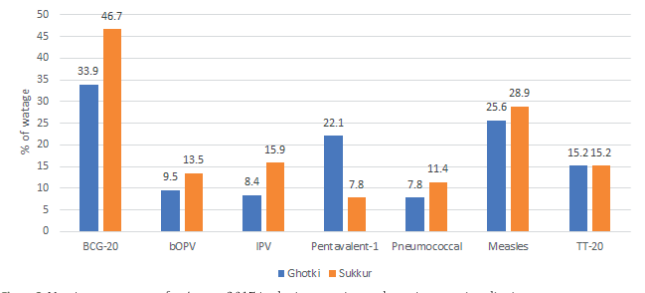
Vaccine wastage rate for August 2017 in the intervention and non intervention districts.

Overall, the vaccine wastage rate was high in the non-intervention Sukkur district compared to the VAN-intervention Ghotki district (**Figure 2**). For example, wastage of BCG was 46.7% in Sukkur compared to 33.9% in Ghotki, OPV was 13.5%in Sukkur compared to 9.5%in Ghotki, and pneumococcal was 11.4%in Sukkur compared to 7.4%in Ghotki. However, the pentavalent wastage was higher in the Ghotki than in Sukkur.

### Quality assurance of data at the district level

Significant data quality issues were observed in the district store in Sukkur in vLMIS reporting, and data was mismatching with what was entered in stock register, along with the entries in registers not being up to date. There were also discrepancies in stock actually available on hand and what was entered in the stock registers. Minor data errors were recorded in receiving from divisional stores and issuance to *taluka* stores. No record of dry stock, such as syringes and safety boxes, was maintained in the stock registers in the district store Sukkur.

### Results of the qualitative interviews

In depth interviews with three key informants (Country Director Deliver Project, Team Leader, VAN Sindh and Director EPI Sindh) were conducted. Key responses are described below.

### Benefits of VAN utilized by relevant authorities

The country director of the project commented that VAN benefits could not be utilized to its fullest because of short project life. EPI director was of the opinion that VAN helped in data analysis and it also aided decision making at district level. EPI director was also of the opinion that the project improved supply chain performance and visibility of data. VAN team leader for Sindh province was of the opinion that VAN improved overall quality of data recording and it helped in timely reporting and uploading of data. He commented that wastage rates decreased, and coverage also improved. VAN Country Director had commented, *“supply chain realization has been brought in Pakistan by the vLMIS/VAN project, and vLMIS and VAN are pioneers of monitoring.”*

### Challenges encountered during implementation

VAN country directed informed that whilst resources were low, but team work and effective management helped. The EPI director was of the view that there were challenges at health facility level and in generating reliable data. EPI Sindh Director had commented, “*receiving of data generated on fortnightly and monthly basis and even at quarterly basis at district and tehsil level was not properly followed*.” VAN team leader of the province commented that limited computer skills of some key staff and internet connectivity hampered timely data uploading and often delayed visibility that was required for decision making. He also pointed out trivial mistakes in the reported data were not corrected despite regular feedback by the monitoring team.

### Willingness of the provincial leadership to scale up

VAN country director believed senior hierarchy of Sindh province was keen to scale up. The EPI director commented that the whole program was chaired by a senior member of national assembly belonging from Sindh province and he oversees the targets and indicators. VAN team leader for Sindh believed Sindh Health Department has been willing to scale-up, but it requires donor support. He had commented that, *“it was for the first time that district officials sat together to analyze the data and took actions as well as made monitoring plans collectively.”*

### Human capacity requirement to scale up

Among the respondents, VAN country director was of the opinion that a 2-3 years long model is required along with engagement of local organizations and QIT master trainers for a sustainable scale up. The EPI director commented that no new recruitment is required and only refreshers can help in scaling up. VAN provincial tea leader was of the opinion that QIT master trainer are key and their presence and involvement will be necessary.

## DISCUSSION

The findings indicate that the VAN project made a positive impact in immunization supply chain management in the intervention district in Pakistan. Having a similar Visibility and Analytics Network (VAN) can help countries worldwide in improving their immunization supply chain management system. This study was unique, as it had both quantitative and a qualitative component. The project concluded two years back but most of the vLMIS operations remained functional. The final evaluation report of the Deliver Project had concluded that the project helped in improving the operation and performance of supply chains. Trends in indicators of supply chain performance, ie, reporting rates, consumption, wastage rates and vaccine coverage, increased significantly in the project-supported provinces and data accuracy helped the managers in making decisions [17].

The benefits of the VAN approach have also been documented in other countries. Kenya’s Ministry of Health, through its National Vaccines and Immunization Programme (NVIP) has embraced the concept of a VAN initiative, as a way to accelerate progress towards its health goals. Kenya envisages that VAN will transform its vaccine supply chain and enhance programme performance [21]. Mozambique’s immunization programme follows a traditional EPI model for immunization supply chain and associated information systems across all provinces. Their model is largely policy driven and implemented through annual plans. In contrast, the VAN Reference Model uses a more fluid approach that adjusts based on what is or is not working at the service-delivery level, as evidenced by an ongoing stream of data [22]. VAN would add a small group of highly skilled workers who are encouraged to make or recommend adjustments based on the data as and when they deem appropriate [22]. A 2018 study from Nigeria documented that the country made remarkable progress in improving performance of immunization supply chain and logistics since the implementation of VAN.[20] VAN approach has moved the supply chain management in Nigeria to the next phase of growth by enabling a culture of data driven decision making [20].

The study researchers could not find recent coverage rates for Ghotki and Sukkur districts. WHO EMRO website reported that current EPI coverage for fully immunized based on Pakistan Demographic Health Survey (2012–2013) and Pakistan Social and Standard Living Measurement (2014–2015) surveys is 65% and 88%, respectively [23]. A real-time comparison can only be made with more recent coverage data for Sukkur and Ghotki districts of Sindh.

The key results of quantitative assessment, such as storage of stock or vaccine wastage, indicate that in the intervention district for both the situation of stock for one month and wastage were better in the intervention district. Vaccine shortages result from higher than expected demand, interruptions in supply or lack of resources to purchase the vaccines [24]. Vaccine wastage is an important factor in calculating vaccine needs. Incorrect calculations can lead to serious vaccine shortages or the programme may not be unable to consume received quantities, leading to increased wastage through expiry [1,24]. It is therefore crucial that all immunization points using vaccines and that the stores handling them monitor their use continuously. Vaccine shortage due to wastage cause critical interruption in immunization services and many children or mothers of the area may not be able to get vaccinated and get the necessary protection against vaccine preventable diseases.

The VAN project was able to establish provincial-level VAN teams and QITs and equip them with the ability to analyze EPI-related data and provide them with the capacity to take actions on a regular basis to incrementally improve the EPI supply chain in the intervention district. This kind of model can be replicated in countries having challenges in supply chain management of vaccines.

The qualitative component of this study focused on gathering information from key informants, mainly about lessons learned and whether intervention can be replicated at a larger scale. Two project-related staff members were of the opinion that it should be replicated in all districts, as limited human resources are required. They also cited short project duration as a key challenge but were of the opinion that VAN helped in producing a critical mass of trained personnel who can be useful as master trainers or at least can be used in the adjacent districts. The benefits of the project cited were zero stock out, decreased wastage rates and improved coverage during the life of the project. The government-related key informant was of the view that projects such as VAN can be replicated but will require long-term donor support. One of the key findings from the qualitative part of the study that should be noted is that there is enough money available in the national immunization support programme and it should be used for vLMIS as well as VAN. There was also a recommendation to pool resources, for example, with the nutrition supply chain.

### Limitations of the study

This research had certain limitations that need to be kept in mind before drawing any conclusion. Only available descriptive results were compared and comments on causality cannot be based on this comparison. Furthermore, it was not possible to control for confounders while selecting districts. The operationalization of variables was also not done. However, even this simple assessment showed that there are benefits in adapting the VAN approach.

## CONCLUSIONS AND RECOMMENDATIONS

VAN helped in improving immunization supply chain management issues in the district in which it was implemented. This suggests that important improvements in the system will be gained by scaling up this project to other districts in Pakistan. The findings of this project have global implications. Countries which are experiencing supply chain management challenges can establish and institute QIT team(s) at national or sub national level which can help the immunization health work force to better manage their data. It is only prudent that there are adequate resources available not just for pilot or demonstration project but beyond so that lessons learned from the pilot phase should be used to scale up VAN nationally.
